# AEIOU strategy for continuous renal replacement therapy in infants <10 kg: a practical and educational framework

**DOI:** 10.3389/fped.2025.1725990

**Published:** 2026-01-09

**Authors:** Mayerly Prada Rico, Jaime Fernández-Sarmiento, Maria Jose Santiago, Francisco Flores

**Affiliations:** 1 Department of Pediatrics and Pediatric Nephrology, Universidad del Bosque, Fundación Cardioinfantil-Instituto de Cardiología, Bogotá, Colombia; 2 Department of Pediatrics and Intensive Care, Universidad de La Sabana, Chía, Colombia; 3 Department of Pediatrics and Intensive Care, Fundación Cardioinfantil-Instituto de Cardiología, Bogotá, Colombia; 4 Pediatric Intensive Care Unit and Pediatrics Department, Hospital General Universitario Gregorio Marañón, Universidad Complutense de Madrid, Madrid, Spain; 5 Professor Pediatric Nephrology and Hypertension, Cincinnati Children’s Hospital Medical Center, Cincinnati, OH, United States

**Keywords:** acute kidney injury (AKI), AEIOU strategy, continuous renal replacement therapy (CRRT), extracorporeal therapies, infants <10 kg

## Abstract

Continuous renal replacement therapy (CRRT) has become a cornerstone in the management of critically ill children with severe acute kidney injury (AKI) and fluid overload. However, its use in neonates and infants weighing less than 10 kg remains particularly challenging due to limited circulating volumes, higher risks of hemodynamic instability, and the scarcity of devices specifically designed for this population. In recent years, new platforms have been introduced, enabling safer extracorporeal support for the smallest patients. Although early experiences suggest that these technologies are feasible and safe, survival outcomes in this fragile group remain suboptimal, and standardized guidelines are still lacking. To address this gap, we propose the AEIOU strategy, an educational and practical framework aimed at organizing and simplifying the essential components required for safe and effective CRRT in infants under 10 kg. *AEIOU* summarizes: A (*Alerts: indications, risks, and contraindications*), E (*Execution Team)*, I (*Inputs: catheters, machines, filters, and solutions*), O (*Orders: prescription components such as priming, anticoagulation, blood and effluent flow rates*), and U (*Unified record: standardized documentation of therapy variables and outcomes)***.** By integrating existing literature with a reproducible mnemonic, AEIOU provides a structured tool for training clinical teams, ensuring quality of care, and laying the foundation for collaborative research. Beyond CRRT, this framework may be adaptable to other extracorporeal therapies in pediatrics. The AEIOU strategy offers a simple yet comprehensive guide to improve implementation, education, and outcome monitoring of CRRT in neonates and small infants.

## 1 Introduction

Continuous renal replacement therapy (CRRT) has become a life-saving intervention for critically ill children with acute kidney injury (AKI) and fluid overload (1, 2). While the use of CRRT is expanding worldwide, delivering these therapies in neonates and infants weighing less than 10 kg remains particularly complex due to small extracorporeal volumes, hemodynamic instability, and the limited availability of dedicated devices (3). The safe and effective use of CRRT therefore requires highly trained multidisciplinary teams and clear educational strategies to standardize practice.

**Figure 1:**
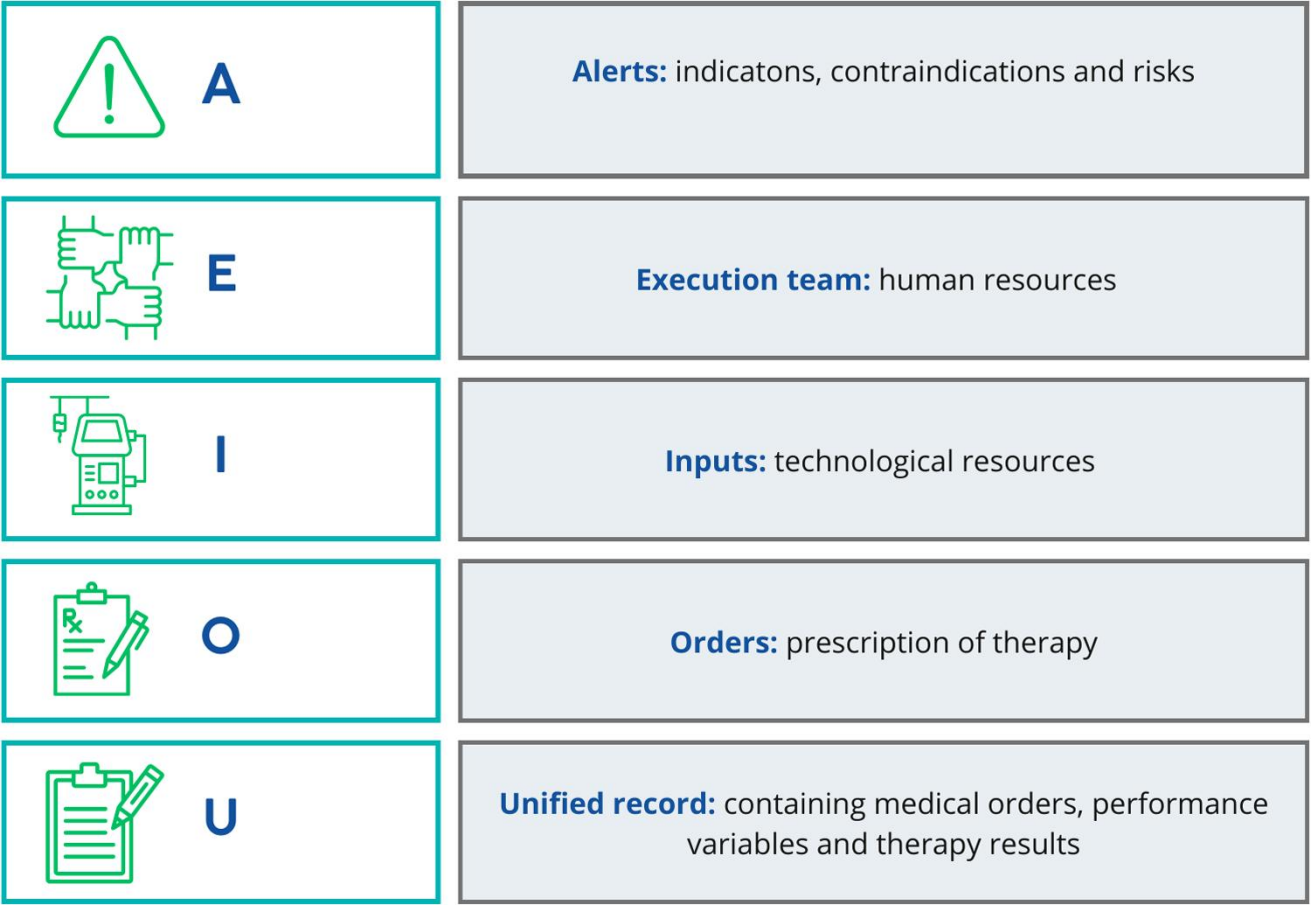
Acronym AEIOU.

To address this need, we propose the *AEIOU strategy*, an educational and practical framework that organizes the essential aspects of CRRT into a simple, reproducible acronym. AEIOU highlights: **A** (Alerts: indications, risks, and contraindications), **E** (Execution Team), **I** (Inputs: catheters, machines, filters, and solutions), **O** (Orders: prescription and technical parameters), and **U** (Unified record: standardized documentation of performance and outcomes) **(**Figure 1**)**. This structured approach is designed to simplify teaching, reduce errors, and improve adherence to best practices, particularly in pediatric intensive care units with limited experience.

In addition, contemporary data from the WE-ROCK cohort reinforce the importance of adopting a structured framework for infants <10 kg receiving CRRT (4). In this study, centers demonstrated substantial practice variability, including more than a tenfold difference in prescribed dosing. This combination of physiologic vulnerability, technical complexity, and wide inter-center variation underscores the need for structured, reproducible frameworks such as AEIOU—tools that can reduce unwarranted variability and promote safer, more consistent care in this high-risk population. The objective of this review is to summarize the current evidence on CRRT in children under 10 kg and to introduce the AEIOU strategy as a practical and educational framework to enhance safety, standardization, and implementation.

## 2 Special considerations for CRRT in infants <10 kg

Acute kidney injury (AKI) affects up to 30% of critically ill neonates admitted to pediatric intensive care units (PICUs) and neonatal intensive care units (NICUs) and is strongly associated with adverse outcomes, particularly when classified as severe AKI (KDIGO stage 2–3) (1, 2). One of the greatest challenges in managing these patients is the provision of renal replacement therapy. Standard hemodialysis or CRRT in this population is complicated by the small circulating blood volume relative to extracorporeal circuit volume, the risks of anticoagulation and bleeding, susceptibility to hypothermia, and the potential for errors in solute and fluid clearance that may be tolerated in adults but carry disproportionate consequences in small infants (3).

Historically, available CRRT platforms were designed for adults, with limited modifications to allow their use in children >10 kg. Over the last two decades, however, devices specifically engineered for infants <10 kg have emerged, including the Aquadex™ FlexFlow System (Nuwellis, USA), CARPEDIEM™ (Cardio-Renal Pediatric Dialysis Emergency Machine), and NIDUS™ (Newcastle Infant Dialysis and Ultrafiltration System). Of these, Aquadex™ and CARPEDIEM™ obtained FDA approval in 2020. These machines incorporate smaller filters and highly precise control of blood (*Q*_b_) and ultrafiltration (*Q*_UF_) flows, thereby reducing hemodynamic instability and technical complications (4).

Early clinical experiences and case series in neonates and infants <10 kg consistently report feasibility and safety, though most studies are limited by small sample sizes (5–14). The central unanswered question remains whether these innovations will translate into improved survival in this highly vulnerable group.

Table 1 summarizes the patient characteristics, technical aspects, and prescription parameters of CRRT therapies reported in studies on the use of new technologies in infants weighing less than 10 kg.

**Table 1:** Summary of patient characteristics, technical aspects, and prescription parameters of CRRT in infants <10 kg.

Category	Study characteristics	Carpediem™			Aquadex™		NiduS™
General Characteristics	Author, year	Vidal et al. (7)	Garzotto et al. (8)	Battista et al. (9)	Askenazi et al. (10)	Menon et al. (4)	Lambert et al. (12)
	*n*	13	26	25	12	117 (72 < 10 kg)	97 (C: 62, I: 35)
	Hours therapy (h)	1,008	2,295	2,125	ND	ND	ND
	Sessions	95	165	131	SD	711 (<10 kg)	ND
	Centers	4 (Italy)	6 (Italy)	5 (France)	1 (EEUU)	3 (EEUU)	6 (UK)
	Body weight	3 kg (IQR 2.5–6.2)	2.9 kg (IQR 2.2–3.6)	3.3 kg (2.5–4.0)	3.4 kg (IQR 3.0–4.3)	4.1 kg (3.1–5.6)	C: 3.2 kg (2.9–3.9) I: 3.7 kg (3.1–5.6)
Indications and Risks	Indication (%)	AKI (46)	FO (85)	FO (60)	CKD (25)	FO (46)	FO or biochemical disturbance
		ESRD (30)	Metabolic or EI (15)	ESRD (20)	AKI (75)	AKI (40)	
		MD (24)		IEM (12)		ESRD (14)	
				EI (8)			
	Diagnosis (%)	ESRD (30.7)	Cardiac (38)	Hypoxic-ischemic encephalopathy (36)	CHD (41.6)	Cardiac (29)	C: Bypass cardiac procedure (48.3), bronchiolitis (1.6), other (50)
		MD (23)	Sepsis (15)	Sepsis (16)	CAKUT (25)	Kidney (43)	I: Bypass cardiac procedure (28.5), bronchiolitis (5.7), other (65.8)
		Cardiopathy (15.3) Other (38.4)	IEM (15)	CKD (20)	Other (33.3)	Other (28)	
			CKD (12)	MD (16)			
			Primary pulmonary disease (12)	Other (12)			
			Others (8)				
	Survived at end CRRT *n* (%)	13 (100)	25 (96.1)	25 (100)	7 (58)	43 (60)	ND
	Survived at hospital discharge *n* (%)	9 (63)	13 (50)	8 (32)	6 (50)	23 (32)	C: 52 (84)
							I: 23 (66)^a^
	Complications *n* (%)	Per session: clotting 3 (3)	Per session: clotting 22 (13), stop therapy for clinical reasons 12 (7), vascular access malfunction 10 (6), technical problems 4 (2.4)	Per session: hemodynamic instability 31 (23), Clotting 25 (19), catheter dysfunction 3 (2), pressure dysfunction 7 (5), restitution failure 4 (3)	Hypothermia: 3/261 days = 11 episodes/1,000 patient-days	Per session: hypothermia 4 (0.6), bleeding 17 (2.39), hypotension 30 (4.2), clot 37 (5.2), thrombocytopenia 2 (0.3), catheter malfunction 12 (1.7)	Hypotensión 1 (2.9)
					Bleeding: 4/261 days = 15 episodes/1,000 patient-days		
Vascular Access	Fr (%)	5 (77)	4 (27)	4.5 (32)	DL: 8 (33.3), 7	6 (35)	NIDUS group without ECMO (*n* = 34):
		6.5 (7.7)	5 (35)	5.5 (24)	(33.3), 6 (8.3)	7–8 (51)	DL: 6.5 (8.8)
		8 (15.3)	6.5 (11)	6 (16)	SL: 2 × 4 (25)	9–10 (7)	SL: 16 (14.7), 18 (35.2), 20 (32), 22 (5.8)
		IJ (77)	7 (3)	6.5 (24)			
				8 (4)			
	Localization (%)	Femoral (23)	IJ (54)	IJ (72%)	IJ (75)	IJ (64)	ND
			Umbilical (11.5)	Subclavian (20)	Femoral (8.3)	Femoral (26)	
			Subclavian (3.5)	Umbilical (4)	IJ + femoral (16.6)	Other (10)	
				Femoral (4)			
Prime	Type (%)	Albumin (92.4)	NS (58)	Per session:	Per circuit (*n* = 101): PRBCs (79)	ECV >10% or hemodynamic instability: PRBCs	ND
		NS (7.6)	Albumin (31) PRBCs (11)	NS (41)	NS (21)	ECV < 10% and hemodynamic stability: crystalloid	
				Albumin or isofundine (52) PRBCs (7)			
Anticoagulation	Anticoagulation (%)	Per patient: heparin (100)	Per session: Heparin (71) NS (29)	Per patient: Heparin (88) NS (12)	Heparin (100)	Per circuit (*n* = 711): Heparin (86.6)	ND
						Citrate (0.28)	
						NS (14.7)	
						Other (0.28)	
Parameters	*Q*_b_ mL/kg/min	5 (IQR 3.8–10.2)	4.5 (IQR 3.4–4.6)	CVVH: 8	10–40 mL/min	10–40 mL/min	ND
				CVVHD: 6			
	Dose	*Q*_d_: 120–600 mL/h	35 mL/kg/h (IQR 28, 42)	*Q*_ef_: 74 mL/kg/h	30 mL/kg/h	24 mL/kg/h or	
			IEM (*n *= 4): 60 mL/kg/h (IQR 52, 69)	(43–99)		2,000 mL/1.73m^2^/h	ND
				*Q*_d_: 600 mL/h			
				(300–600)			
	Modality (%)	CVVHD (100)	CVVH (100)	CVVH (80)	CVVH (100)	CVVH (67)	CVVH
				CVVHD (20)		SCUF (12)	
						Prolonged intermittent KRT (21)	

ND, NO Date; C, control; I, intervention; MD, metabolic disease; FO, fluid overload; ESRD, end-stage renal disease; AKI, acute kidney injury; IEM, innate errors of metabolism; EI, electrolyte imbalances CKD, chronic kidney disease; AKI, acute kidney injury; CHD, congenital heart disease; CAKUT, congenital abnormalities kidney urinary tract; CRRT, Continuous renal replacement therapy; DL, Double Lumen; SL, single lumen; IJ, internal jugular; ECMO, extracorporeal membrane oxygenation; NS, normal saline solution; PRBCs, packed red blood cells; ECV, extracorporeal volume; NA, none anticoagulation; *Q*_b_, blood flow rate; *Q*d, dialysate flow rate: *Q*r, replacement fluid rate; *Q*ef, total effluent rate; IEM, innate errors of metabolism; CVVH, continuous veno-venous hemofiltration; CVVHD, continuous veno-venous hemodialysis; CVVHDF, continuous veno-venous hemodiafiltration; SCUF, slow continuous ultrafiltration; KRT, kidney remplacement therapy.

a Survived at PICU (pediatric intensive care unit) discharge.

Recent multicenter efforts, such as the ICONIC consortium, represent a critical step forward. By systematically collecting clinical and outcome data in infants treated with CARPEDIEM™, ICONIC seeks to define best practices and inform standardized treatment protocols (12). Nevertheless, there are currently no universally accepted guidelines or consensus statements for CRRT in infants <10 kg.

Collaborative registries, such as WE-ROCK (Worldwide Exploration of Renal Replacement Outcomes Collaborative in Kidney Disease), have reported modest improvements in survival among critically ill children weighing less than 10 kg who require CRRT, compared with historical cohorts. However, mortality rates and major adverse kidney events remain unacceptably high (14, 15). In this context, structured frameworks such as the AEIOU strategy may provide clinicians with a practical tool to organize key considerations and promote safe, consistent CRRT delivery, while the pediatric nephrology and critical care communities continue to build the evidence base needed to inform future guidelines.

### 2.1 A: alerts

#### 2.1.1 Objectives

The primary objective of this phase is to ensure the timely recognition of clinical scenarios warranting CRRT in infants weighing <10 kg, including severe AKI, diuretic-resistant fluid overload, life-threatening electrolyte or acid–base disturbances, metabolic diseases, and selected intoxications, while simultaneously identifying the therapy's inherent risks and implementing appropriate safety precautions.

#### 2.1.2 Indications

Technologies designed to deliver CRRT in infants weighing less than 10 kg have been employed in critically ill neonates and infants admitted to the PICU for both renal and non-renal indications. Renal indications include severe AKI with refractory uremia, hyperkalemia, or metabolic acidosis unresponsive to conventional therapy. Non-renal indications encompass fluid overload (FO), hyperammonemia, and intoxications with dialyzable substances, all of which represent the most frequent reasons for initiating CRRT in this population.

Among these, FO is the leading indication for CRRT initiation in infants <10 kg (Table 1). A recent secondary analysis from the WE-ROCK cohort included 210 patients under 10 kg who received CRRT for AKI or FO across 32 centers in seven countries (14). In this subgroup, the median FO at CRRT initiation was 16.4% (IQR 6.3–32.8). By comparison, when analyzing the entire cohort of patients <25 years, the median FO was 7.4% (IQR 2.4–18.1) (15), a value lower than previously reported in the literature (16). This observation suggests a shift in clinical reasoning toward initiating CRRT at earlier stages of FO, reflecting the well-recognized negative impact of FO on survival and renal recovery in critically ill children (17, 18).

#### 2.1.3 Risks

Complications associated with CRRT in infants <10 kg vary across studies but commonly include hypotension (7%–23%), thrombocytopenia (22%), and circuit clotting (13%–19%) (Table 1), and despite technological advances, survival remains modest: the ppCRRT registry reported 56% mortality at PICU discharge among 84 patients <10 kg (16), while the more recent WE-ROCK cohort showed a survival of 46.1% (14). Beyond these outcomes, infants <10 kg exhibit a distinct physiologic vulnerability that heightens procedural risk. Vascular access complications are frequent due to catheter–vessel caliber mismatch; in the London cohort, insertion-site bleeding occurred in 6.7% of sessions and hypotension requiring intervention in 11.9% (3).

Metabolic instability—including rapid electrolyte shifts, acid–base disturbances, and hypothermia—arises from blood priming requirements and the disproportionate extracorporeal volume relative to circulating blood volume. In the Aquadex™ infant series, transient hypothermia, puncture-site bleeding, systemic bleeding, and occasional atrial thrombosis were noted, underscoring the hemostatic fragility of infants <5 kg (10). Collectively, these data highlight that infants <10 kg face converging hemodynamic, metabolic, and access-related risks that are intrinsic to critical illness and magnified by the technical challenges of extracorporeal support, reinforcing the need for structured frameworks that can more effectively “alert” clinicians to early risk signals and optimize timing of CRRT initiation.

#### 2.1.4 Contraindications

The success of CRRT in infants critically depends on secure vascular access, and the inability to obtain adequate access due to severe bleeding, vascular thrombosis, infection, or extensive skin injury remains the only absolute contraindication to initiating therapy (20–22). In all other situations, contraindications are relative and context-dependent, including active bleeding with high transfusion requirements, profound hemodynamic instability not responsive to vasoactive support, or institutional limitations such as lack of neonatal-specific technology or insufficiently trained personnel, all of which may compromise safety. Importantly, most conditions traditionally viewed as barriers should not be interpreted as absolute exclusions (23); instead, they demand a careful multidisciplinary risk–benefit assessment, particularly in infants <10 kg where therapeutic margins are exceptionally narrow. This nuanced approach underscores the need for specialized training, structured frameworks such as the AEIOU strategy, and regional collaboration to expand access to CRRT while minimizing complications.

### 2.2 E: execution team

#### 2.2.1 Objectives

The safe and effective delivery of CRRT in infants <10 kg requires a multidisciplinary team supported by appropriate technological and institutional resources. Beyond physicians and specialized nursing staff, essential contributors include pharmacy services, clinical laboratories, and biomedical engineering, all of whom ensure therapy safety and continuity (4). Updated institutional documents such as policies, standard operating procedures, and evidence-based clinical practice guidelines are equally critical to standardize care, minimize variability, and promote adherence to best practices. Together, these human, technological, and documentary resources form the backbone of a high-quality CRRT program in the PICU.

#### 2.2.2 Program leadership and standardization

The successful implementation of a CRRT program for infants under 10 kg requires the designation of a dedicated program leader responsible for coordinating the multidisciplinary team, developing and updating standardized protocols, and ensuring uniform adherence to evidence-based practices. This leader—typically a pediatric intensivist or nephrologist with expertise in extracorporeal therapies—should oversee team training, protocol audits, and outcome monitoring, while fostering communication and collaboration among all participating members, including nursing, pharmacy, and biomedical engineering. The presence of a defined leadership structure promotes consistency across shifts and providers, minimizes variability in practice, and strengthens the overall quality and safety of CRRT delivery.

#### 2.2.3 Medical and nursing teams

The delivery of CRRT in infants <10 kg requires close collaboration between pediatric intensivists, neonatologists, and pediatric nephrologists. While intensivists often lead the management in the PICU, the participation of nephrologists adds expertise in prescription and troubleshooting. In patients with difficult vascular anatomy or in very small infants, interventional radiologists or pediatric surgeons may be required to secure central venous access. Specialized nursing staff are equally essential, responsible for priming, assembling, and monitoring the circuit, as well as ensuring consistent delivery of the therapy. Ideally, nurse-to-patient ratios should be at least 1:1, although surveys indicate that this standard is not always met.

The multinational ESPNIC survey highlighted the variability of CRRT practice in Europe: intensivists were the primary prescribers in 70% of centers, nephrologists in 12%, and only 4% reported joint responsibility (20). Nursing teams in most centers were primarily in charge of machine operation and circuit management. Importantly, the survey also revealed that in the majority of cases (56%) the nurse-to-patient ratio was 1:1, while 24% reported 2:1 and only 18% of providers cared for more than one patient simultaneously. These findings underscore the heterogeneity in workforce allocation and the challenges in maintaining optimal staffing in high-demand settings.

Given that CRRT in infants <10 kg is still infrequent in many PICUs, ongoing training is crucial to maintain competence. Structured educational programs should combine theoretical modules, bedside supervision, simulation-based training, and access to digital resources. Although no universal certification standards currently exist, periodic education and skills reinforcement are essential. Notably, in the ESPNIC survey, only 61% of nurses reported having received formal training, and nearly one-third of PICUs lacked requirements for certification or recertification. These findings highlight a major gap and reinforce the need for standardized curricula and continuous professional development.

#### 2.2.4 Competencies and safety processes

To operate CRRT safely in infants <10 kg, teams must demonstrate core competencies that include mastery of circuit priming with minimal extracorporeal volume exposure, troubleshooting of pressure alarms and flow instability, early recognition of access dysfunction, precise titration of anticoagulation strategies, and rapid interpretation of laboratory trends that signal metabolic or hemodynamic compromise. These skills must be supported by structured processes such as pre-CRRT safety checklists (confirming catheter position, machine calibration, anticoagulation plan, and fluid targets), standardized bedside monitoring bundles, and post-connection stabilization protocols. Embedding these competencies within routine workflows strengthens reliability and reduces preventable errors—an essential goal in a population with extremely narrow safety margins.

#### 2.2.5 Pharmacy team

Pharmacists play a vital role in ensuring patient safety during CRRT. They provide guidance on dose adjustments for medications impacted by extracorporeal clearance based on estimated CRRT dose provided and drug carachteristics. In some centers, pharmacists are also involved in preparing dialysis fluids or anticoagulation solutions, which is particularly relevant in small infants where fluid composition must be precisely tailored. This collaboration minimizes medication errors and ensures individualized therapy, which is particularly relevant given that most centers are currently using commercially based CRRT solutions.

#### 2.2.6 Clinical laboratory team

Laboratory support is essential to guide real-time adjustments during CRRT. Monitoring acid–base balance, electrolyte homeostasis, and coagulation parameters informs critical prescription changes and allows rapid response to complications. Continuous availability of laboratory services during therapy is therefore indispensable to maintain patient safety and to optimize treatment efficacy.

### 2.3 I: inputs

#### 2.3.1 Objective

To ensure the safe and effective delivery of CRRT in infants <10 kg by selecting and optimizing technological resources (vascular catheters, machines, filters, extracorporeal circuits, and solutions) that minimize extracorporeal volume, reduce complications, and adapt therapy to the physiological needs of this vulnerable population.

#### 2.3.2 Catheters

Except for NIDUS™, which operates with a single-lumen central venous line, all other neonatal CRRT systems require a double-lumen catheter to maintain adequate blood flow. Conventional devices often require catheters ≥7 Fr, while CARPEDIEM™ can be used with 4 Fr catheters, an important advantage in neonates. Reports of using two single-lumen catheters exist, but very small devices carry risks of thrombosis, poor flow, and malfunction (8, 9). Dedicated dialysis catheters are designed with sufficient rigidity to resist pump pressures and with lengths that optimize tip positioning.

The right internal jugular vein has become the preferred access due to favorable anatomy and reduced recirculation (21). In WE-ROCK, this site was used in 66% of cases, compared with 16% in ppCRRT; femoral access decreased from 73% in ppCRRT to 30% in WE-ROCK, while subclavian use remained rare (<10%) (15, 22).

#### 2.3.3 Machines

Neonatal CRRT platforms have been specifically engineered to address the unique physiological vulnerabilities of infants <10 kg by minimizing extracorporeal circuit volume, enhancing ultrafiltration precision, and permitting exceptionally low blood-flow rates. Among these devices, CARPEDIEM™—FDA-approved for infants 2.5–10 kg (5–9)—and the HF-20 hemofilter for Prismaflex™, FDA-approved for children 8–20 kg (4), represent the only platforms with regulatory authorization for use in this population; both also hold CE Mark approval in Europe, supporting their broader international adoption. Evidence from retrospective comparisons shows that infants <5 kg treated with CARPEDIEM™ had higher survival during therapy than historical ppCRRT controls treated with standard machines, which required substantially higher vasoactive support at initiation—highlighting the hemodynamic burden of non-neonatal platforms (6–12, 19).

In contrast, Aquadex™, although FDA-approved only for patients >20 kg, has been used off-label in selected infants because of its low extracorporeal volume (33 mL) and flexible blood-flow range (5–40 mL/min), enabling treatment of children as small as 3 kg (10, 11). The NIDUS™ system, employing a syringe-driven circuit with extracorporeal volumes as low as 17 mL, is CE-approved for neonatal use in Europe and has demonstrated feasibility in neonates as small as 800 g (12). While Aquadex™ and NIDUS™ allow hemofiltration and ultrafiltration, CARPEDIEM™ is the only miniaturized platform that offers hemodialysis capability (Table 2).

**Table 2:** Comparison of new technologies for CRRT in <10 kg.

Machine	ECV (mL)	Filter (m^2^)	*Q*_b_ (mL/min)	Infusion rates (mL/h)	Modality	Dialysis or replacement fluid
Prismaflex™	60	2	10–100	0–500	CVVH CVVHD CVVHDF SCUF	PrismaSATE™
						(Gambro Renal Products)
Aquadex™	33	12	10–40	0–500	CVVH	Prismasol or phoxillum
					SCUF	(Gambro Renal Products)
CARPEDIEM™^a^	27	75	2–50	0–150 (dialysate)	CVVH	HBioFluid 2.5
					SCUF	
	32	75		0–600 (replacement)	CVVH	(Haemiopharm biofluids S.R.L)
					CVVHD SCUF	
	41	25		0–900 (ultrafiltration)		
NIDUS™	<17	45	5	0–60	CVVHD	ND
					SCUF	

ECV, extracorporeal volume; *Q*_b_, blood flow; UF, ultrafiltration; CVVH, continuous veno-venous hemofiltration; CVVHD, continuous veno-venous hemodialysis; CVVHDF: continuous veno-venous hemodiafiltration; SCUF: slow continuous ultrafiltration; ND: No date.

a Infusion rates represent the maximum programmable flow of the dialysate, replacement, and ultrafiltration pumps, respectively, as specified by the manufacturer. These values do not correspond to clinical net ultrafiltration limits, which are determined according to patient condition and usually remain below 20% of blood flow per hour. Total fluid removal should not exceed 2,000 grams per day. Modified from: Mohamed TH, et al. (4).

#### 2.3.4 Dialysis or replacement fluid

Most solutions approximate physiologic electrolyte concentrations, with variable potassium, calcium, and bicarbonate content. Bicarbonate-buffered solutions are preferred over lactate-based fluids for greater hemodynamic stability and improved metabolic control. When regional citrate anticoagulation is used, calcium-free solutions with lower bicarbonate concentrations help prevent metabolic alkalosis resulting from citrate metabolism. The CARPEDIEM™ system, however, offers only a single solution containing calcium and a high bicarbonate concentration.

### 2.4 O: orders

#### 2.4.1 Objective

To define the essential components of CRRT prescription in infants <10 kg, ensuring individualized, safe, and effective therapy through precise programming of extracorporeal circuits, anticoagulation strategies, and machine parameters.

#### 2.4.2 Circuit priming

Circuit priming can be performed with red blood cells, crystalloids, or colloids such as albumin. Red blood cell priming may be indicated when the extracorporeal volume (ECV) exceeds 10% of the patient's blood volume, or in cases of anemia or hemodynamic instability. However, this approach carries risks including electrolyte disturbances (hypocalcemia, hyperkalemia), acid–base disorders, thrombocytopenia, coagulopathy, and vasoplegia. A major advantage of neonatal-specific CRRT machines is their ability to maintain ECV below 10% in most cases, thereby minimizing the need for blood priming.

The following formula is used to determine the ECV:

$$\mathrm{ECV}\;(\text{\%})=\frac{\mathrm{ECV}\;(\mathrm{mL})}{\mathrm{Weight}\;(\mathrm{kg})}\times 80\;(\mathrm{mL}/\mathrm{kg})*$$

*In neonates, the value should be multiplied by 100 (mL/kg).

#### 2.4.3 Anticoagulation

Effective anticoagulation is essential to prevent clotting of the extracorporeal circuit, as inadequate anticoagulation reduces therapy efficacy, increases blood loss, and increases nursing workload (24). Systemic heparin remains widely used, typically administered pre-filter at 10–20 IU/kg/h with or without an initial bolus, targeting a partial thromboplastin time 1.5–2× baseline while monitoring platelets given the 1%–5% risk of heparin-induced thrombocytopenia (25). However, systemic anticoagulation carries a substantial bleeding risk (10%–50%), particularly after surgery, trauma, mucosal injury, or in patients with underlying coagulopathy (26–28).

Regional citrate anticoagulation (RCA) has become the preferred modality in many neonatal and infant programs because its anticoagulant effect is confined to the circuit. Citrate chelates calcium pre-filter to inhibit thrombin formation, while the calcium–citrate complex is removed by dialysis or metabolized to bicarbonate; systemic calcium is replaced post-filter. RCA reduces bleeding complications compared with systemic heparin (26–28). The most common solutions include ACD-A—typically infused at 1.5× blood flow to maintain in-filter ionized calcium <0.4 mmol/L—and Prismocitrate, titrated based on ionized calcium levels.

Data from the WE-ROCK cohort show that RCA was the most frequently used anticoagulation strategy in infants <10 kg (52%), followed by heparin (31%), no anticoagulation (10%), and other agents (7%) (13). By contrast, early CARPEDIEM™ studies reported more frequent heparin use, likely reflecting limited initial experience with citrate in these newer platforms (Table 1). In contemporary practice, centers such as Cincinnati Children's Hospital now use RCA in approximately 50% of CARPEDIEM™ circuits (this observation is based on personal communication with the program leadership).

Beyond heparin and citrate, prostacyclin (epoprostenol) and nafamostat serve as alternatives in selected high-risk infants. Prostacyclin provides circuit-directed anticoagulation and has demonstrated mean circuit lives of ∼50 h in acute liver failure without major bleeding, supporting its use when heparin is contraindicated (29, 30). Nafamostat, a short-acting serine-protease inhibitor, offers potent regional anticoagulation with minimal systemic effects; in a two-center cohort (158 patients, 455 filters), it achieved circuit life comparable to RCA (median 38 vs. 36 h) with similar bleeding risk and without citrate-related metabolic complications, including in patients with liver dysfunction (31). Experience with these agents in infants <10 kg remains limited to specialized centers, and their use should be individualized according to bleeding risk, local expertise, and drug availability.

#### 2.4.4 Blood flow rates (*Q*_b_)

Unlike older children, in whom *Q*_b_ during CRRT are typically set around 5 mL/kg/min, infants weighing <10 kg often require higher flows (10 mL/kg/min). This adjustment is dictated by vascular access size and the characteristics of the device employed. When using machines originally designed for adults, minimum operational flows are usually ≥60 mL/min (with HF20 minimal blood flow rate is 20 mL/min), which translates into 10–15 mL/kg/min in a small infant. In contrast, neonatal-specific technologies can operate safely at *Q*_b_ as low as 20 mL/min, providing greater flexibility and hemodynamic tolerance in this population. Data from the WE-ROCK cohort confirm this practice, reporting a median *Q*_b_ of 8 mL/kg/min (IQR 5.9–11.3) among infants <10 kg (14). Table 1 summarizes *Q*_b_ parameters reported in studies of children treated with new-generation devices.

#### 2.4.5 Dose

KDIGO guidelines recommend CRRT delivered doses of 20–25 mL/kg/h in adults, although higher prescriptions (30–35 mL/kg/h) are often used to offset therapy interruptions (21). In pediatrics, no large-scale trials define the optimal dose, and practice remains heterogeneous.

In the WE-ROCK cohort, the mean prescribed dose across the entire population was 41.9 mL/kg/h (IQR 30.9–59.9), with 55% receiving >40 mL/kg/h. In infants <10 kg, the mean dose was substantially higher: 63.8 mL/kg/h (IQR 49.2–88.1), with 85% receiving >40 mL/kg/h. Marked inter-center variability was reported, with prescriptions ranging from 27.8 to 187.8 mL/kg/h (14).

#### 2.4.6 Modality

The choice of modality depends on the device and clinical context. New pediatric-specific platforms (CARPEDIEM™, Aquadex™, NIDUS™) allow for continuous venovenous hemofiltration (CVVH), hemodialysis (CVVHD), or hemodiafiltration (CVVHDF), with flexibility to individualize therapy. International surveys of European PICUs demonstrated CVVHDF as the most frequent modality (51%) (20). Similarly, in WE-ROCK, CVVHDF predominated in both the overall cohort (76%) and in infants <10 kg (69%) (14, 15). This preference reflects the ability of CVVHDF to optimize solute clearance while maintaining fluid control, particularly relevant in critically ill neonates with multiorgan dysfunction.

#### 2.4.7 Ultrafiltration flow (Q_UF_)

Q_UF_ represents the total ultrafiltrate removed per unit of time, comprising net ultrafiltration (UF_NET_) and, depending on modality, replacement or dialysate flows (29). Fluid removal is guided by clinical status, fluid balance, and degree of fluid overload. In general, net removal >5% of body weight is not recommended, and with CARPEDIEM™, hourly ultrafiltration should remain <20% of blood flow (32). Modern neonatal platforms incorporate highly precise weighing systems, capable of detecting errors as low as 1 g/h, a critical safeguard in this population where even small discrepancies can have deleterious hemodynamic or metabolic consequences. Additionally, for CARPEDIEM™, manufacturer specifications list infusion rates of 0–150, 0–600, and 0–900 mL/h for the dialysate, replacement, and ultrafiltration pumps, respectively. These parameters indicate pump capabilities rather than prescribed net ultrafiltration limits.

In summary, CRRT dosing and prescription in infants <10 kg remain an evolving field. Observational data suggest higher prescribed doses and a predominance of CVVHDF, with RCA as the preferred anticoagulation strategy. While newer devices mitigate technical limitations by reducing extracorporeal volume and enhancing ultrafiltration precision, practice variation persists. Standardization through multicenter collaboration and prospective studies will be essential to define optimal dosing strategies and improve outcomes in this fragile population (33, 34).

### 2.5 U: unified record

#### 2.5.1 Objective

To establish a standardized, accessible, and integrated documentation system that improves safety, optimizes clinical decision-making, and facilitates the generation of high-quality data for research and quality improvement in pediatric CRRT.

The development of a CRRT program in infants <10 kg must extend beyond protocols and guidelines to include the systematic implementation of a unified record. This record should consolidate all aspects of prescription, delivery, monitoring, and outcomes into a practical tool, ideally embedded within the electronic medical record. Its structured completion functions as a safety checkpoint before, during, and after therapy, ensuring verification of medical orders, early identification of complications, and timely adjustment of prescriptions. Moreover, the unified record generates reliable longitudinal data that form the foundation for clinical audits, benchmarking across institutions, and the design of multicenter research initiatives. By standardizing documentation, programs not only enhance patient safety and quality of care but also strengthen the evidence base for CRRT in the smallest and most vulnerable patients (Table 3).

**Table 3:** AEIOU approach to CRRT in infants weighing <10 kg.

AEIOU Approach to CRRT in Infants Weighing <10 kg		
A: Alerts		
Indications	Contraindications	Risks
Fluid overload	Inability to secure vascular access	Hypotension
Refractory uremia, hyperkalemia, or metabolic acidosis,	Ethical dilemmas (futile treatment)	Hypothermia
Oliguria	Restrictions on access to technology,	Electrolyte imbalance
Dialyzable molecule intoxication	supplies, or absence of an expert group	Hemorrhage
IME (hyperammonemia)		Coagulation or circuit failure
		Thrombocytopenia

E: Execution Team	
Medical and nursing staff	Other
Neonatologist or pediatric intensivist	Pharmaceutical
Pediatric nephrology specialist	Laboratory
Interventional radiologist and/or pediatric surgeon	Biomedical engineering
Specialized nurse (2:1 or 1:1)	

I: Inputs			
Vascular Access	Machine	Dialysis or replacement fluid	Filter (m^2^)
DLC, 4 Fr^b^	Low ECV devices	Depending on the chosen technology, bicarbonate base, phosphorus-free, and electrolyte concentrations that approximate physiological levels	0.045–0.2
DLC, 7 Fr^a^	Low blood flow rates and minimal UF discrepancies		VEC: 17–60 mL
	Prismaflex, HF20™, CARPEDIEM™, Aquadex™, NIDUS		

O: Orders					
Circuit priming	Anticoagulation	Modality	*Q*_b_	Dose	*Q*_UF_
ECV <10%: normal saline	Heparin:Bolus: 10–20 IU/kg; infusion: 10–20 IU/kg/h; target: PTT 1.5 to 2.0 times the normal range	CVVH	10–15 mL/kg/min^a^	35–60 mL/kg/h	UF total
ECV >10%: 5% albumin or red blood cells	Citrate: Dose adjusted to maintain intrafilter Ca_i_ levels below 0.4 mmol/L	CVVHD	5–10 mL/kg/min^b^		CVVH, CVVHDF = *Q*r + UF_NET_
		CVVHDF			CVVHD and SCUF = UF_NET_
		SCUF			
U: Unified Record					
Patient demographic and clinical data, supplies (filter size, catheter type), prescription details (modality, anticoagulation, priming, dose, and blood flow rate), and therapy outcome variables.					

IME, innate metabolic error; DLC, double lumen catheter; ECV, Extracorporeal volume; UF, ultrafiltration; PTT, partial thromboplastin time; Cai, ionized calcium; CVVH, continuous veno-venous hemofiltration; CVVHD, continuous veno-venous hemodialysis; CVVHDF, continuous veno-venous hemodiafiltration; SCUF, Slow continuous ultrafiltration; *Q*_b_, blood flow.

a With conventional technologies.

b With novel technologies.

## 3 Implementation and application of the AEIOU framework

Successful adoption of the AEIOU strategy in centers caring for infants <10 kg requires deliberate integration of the framework into institutional workflows, guided by principles of implementation science. First, programs should engage key stakeholders—including intensivists, nephrologists, nursing leadership, pharmacy, and biomedical engineering—to ensure shared understanding of the framework's purpose and applicability. Readiness assessments can help identify local barriers, such as limited experience with neonatal CRRT, gaps in training, or absence of standardized protocols.

Second, centers may embed AEIOU into clinical practice by developing unit-specific checklists, flow diagrams, and order templates that reflect each domain of the framework. Simulation-based training and structured onboarding modules allow teams to apply AEIOU in controlled environments, reinforcing competencies and increasing reliability. Incorporating AEIOU elements into electronic health records—such as unified CRRT order sets or automated documentation prompts—can further enhance adherence and reduce unwarranted practice variation.

Third, programs should establish audit and feedback cycles, using metrics such as time-to-initiation, catheter-related complications, circuit lifespan, anticoagulation errors, and adherence to AEIOU components as part of continuous quality improvement. These data also support benchmarking and multicenter collaboration, enabling centers to track performance over time and identify opportunities for refinement.

## 4 Limitations

Although the AEIOU strategy provides a structured and pragmatic framework to organize the key elements of CRRT delivery in infants under 10 kg, several limitations must be acknowledged. First, AEIOU is not derived from prospective clinical validation but rather from expert synthesis of available evidence and practice-based experience; therefore, its applicability may vary across institutions with different resources and case volumes. Second, while the mnemonic simplifies complex processes into five domains, it may risk oversimplification, potentially overlooking patient-specific nuances such as comorbidities, pharmacologic interactions, or unique technical challenges. Third, the lack of universally standardized guidelines for CRRT in this population means that AEIOU should be regarded as a complementary educational and organizational tool, rather than a substitute for formal training or individualized clinical judgment. Finally, the effectiveness of AEIOU in improving patient outcomes remains to be demonstrated through multicenter implementation studies and integration into quality improvement initiatives.

## 5 Opportunities and future directions in CRRT for infants <10 kg

The AEIOU framework offers multiple opportunities to advance the quality, safety, and standardization of CRRT delivery in infants <10 kg. A key future direction is the prospective validation of the strategy across diverse centers, examining its impact on time-to-initiation, circuit longevity, anticoagulation safety, and clinically meaningful outcomes. Incorporating AEIOU into simulation curricula, structured onboarding programs, and electronic health record pathways could enhance adherence, reduce practice variability, and support consistent decision-making in high-risk infants. The framework also provides a foundation for multicenter quality improvement collaboratives, enabling benchmarking of process metrics and facilitating shared learning. As neonatal CRRT technologies continue to evolve, AEIOU can serve as a dynamic, adaptable scaffold—integrating new devices, anticoagulation approaches, and monitoring capabilities while maintaining a unified logic for bedside application. Ultimately, the future of CRRT in infants <10 kg will depend not only on technological innovation, but on the systematic implementation, refinement, and evaluation of structured strategies such as AEIOU that translate evidence into reliable, reproducible practice.

## 6 Conclusion

Continuous renal replacement therapy in infants weighing less than 10 kg remains one of the most demanding areas of pediatric critical care, where technological advances alone are insufficient to guarantee safe and effective therapy. The AEIOU strategy—Alerts, Execution Team, Inputs, Orders, and Unified Record—provides a structured, evidence-informed framework that translates existing knowledge into consistent bedside practice. By guiding early recognition of CRRT indications, clarifying team competencies, standardizing the selection of machines and solutions, organizing prescription principles, and promoting unified documentation, AEIOU serves as a practical tool to reduce variability and strengthen clinical decision-making in this vulnerable population.

Future progress in the field will depend on the prospective evaluation and iterative refinement of the AEIOU framework across diverse institutions, its integration into training curricula and quality-improvement initiatives, and its use as a scaffold for multicenter collaboration. Ultimately, the advancement of CRRT in infants <10 kg will require not only continued innovation in miniaturized technologies, but also the widespread adoption of structured strategies such as AEIOU that ensure safer, more reliable, and more equitable care for the smallest and most fragile patients.
